# Peripheral Infection after Traumatic Brain Injury Augments Excitability in the Perilesional Cortex and Dentate Gyrus

**DOI:** 10.3390/biomedicines9121946

**Published:** 2021-12-19

**Authors:** Ying Wang, Pedro Andrade, Asla Pitkänen

**Affiliations:** 1 A. I. Virtanen Institute for Molecular Sciences, University of Eastern Finland, P.O. Box 1627, FI-70211 Kuopio, Finland; wangying@dmu.edu.cn (Y.W.); pedro.andrade@uef.fi (P.A.); 2 Department of Neurology, The First Affiliated Hospital of Dalian Medical University, Dalian 116001, China

**Keywords:** c-Fos, early gene activation, epileptogenesis, lipopolysaccharide, pentylenetetrazole, post-traumatic epilepsy, seizure susceptibility, traumatic brain injury

## Abstract

Peripheral infections occur in up to 28% of patients with traumatic brain injury (TBI), which is a major etiology for structural epilepsies. We hypothesized that infection occurring after TBI acts as a “second hit” and facilitates post-traumatic epileptogenesis. Adult male Sprague–Dawley rats were subjected to lateral fluid-percussion injury or sham-operation. At 8 weeks post-injury, rats were treated with lipopolysaccharide (LPS, 5 mg/kg) to mimic Gram-negative peripheral infection. T2-weighted magnetic resonance imaging was used to detect the cortical lesion type (small focal inflammatory [TBI_FI_] vs. large cavity-forming [TBI_CF_]). Spontaneous seizures were detected with video-electroencephalography, and seizure susceptibility was determined by the pentylenetetrazole (PTZ) test. Post-PTZ neuronal activation was assessed using c-Fos immunohistochemistry. LPS treatment increased the percentage of rats with PTZ-induced seizures among animals with TBI_FI_ lesions (*p* < 0.05). It also increased the cumulative duration of PTZ-induced seizures (*p* < 0.01), particularly in the TBI_FI_ group (*p* < 0.05). The number of c-Fos immunopositive cells was higher in the perilesional cortex of injured animals compared with sham-operated animals (*p* < 0.05), particularly in the TBI-LPS group (*p* < 0.05). LPS treatment increased the percentage of injured rats with bilateral c-Fos staining in the dentate gyrus (*p* < 0.05), particularly in the TBI_FI_ group (*p* < 0.05). Our findings demonstrate that peripheral infection after TBI increases PTZ-induced seizure susceptibility and neuronal activation in the perilesional cortex and bilaterally in the dentate gyrus, particularly in animals with prolonged perilesional T2 enhancement. Our data suggest that treatment of infections and reduction of post-injury neuro-inflammation are important components of the treatment regimen aiming at preventing epileptogenesis after TBI.

## 1. Introduction

Approximately 2.5 million people in both Europe (www.center-tbi.eu/ accessed on 23 November 2021) and the United States (www.cdc.gov/traumaticbraininjury accessed on 22 November 2021) experience traumatic brain injury (TBI) each year. The risk of epileptogenesis increases according to the severity of the TBI: approximately 2- to 4-fold after mild, 8-fold after moderate, and 16-fold after severe TBI [1,2,3]. Up to 53% of patients with penetrating TBI develop epilepsy [4,5]. TBI causes 10% to 20% of symptomatic epilepsy and 5% of all types of epilepsy [6,7]. Despite the large number of epidemiologic studies reporting risk factors for epileptogenesis after TBI [8], the factors and events occurring over the lifetime of a given subject that lead to post-traumatic epilepsy (PTE) remain largely unknown.

The evolution of post-traumatic epileptogenesis overlaps with the progression of secondary brain damage, which can continue for days to weeks to months after TBI and includes neuroinflammation, oxidative stress, excitation-inhibition imbalance, and blood–brain barrier damage, as well as synaptic and network plasticity alterations [9]. Some of these molecular and cellular changes contribute to post-traumatic epileptogenesis, whereas others can support spontaneous recovery [10]. To date, the effects of additional events modulating secondary damage during the post-TBI aftermath have received little attention, even though their prevention and/or treatment could present an important avenue for mitigating post-traumatic epileptogenesis.

The concept of “microglia priming,” in which “the brain is primed by chronic central nervous system (CNS) diseases to show exaggerated responses to a subsequent hit, which induces an inflammatory response, whether systemic or central in origin,” was introduced by Combrinck et al. [11]. Activation of innate inflammatory pathways is an elementary component of the secondary injury in both experimental and human TBI [12]. In animal models, the inflammatory response is most robust during the 1 to 3 weeks post-TBI, but microglia can remain activated in the brain for months [13]. A positron emission tomography (PET) imaging study in humans with TBI using [11C] (R)PK11195 demonstrated that microglial activation can last up to 17 years, and is associated with cognitive decline [14].

Bacterial infections represent important post-TBI secondary hits as they commonly occur but seem innocuous because they are treatable by existing medications. Up to 50% of severe TBI patients have been suggested to suffer from infections during their hospital stay, and infection-related mortality can be as high as 28% [15,16,17,18,19,20]. Infections are most common in patients with the lowest Glasgow Coma Scale scores, that is, in patients with the highest risk of epileptogenesis [18]. To date, no evidence for a clear association between TBI-related infections and PTE has been reported, which may relate to relatively small study populations and short follow-up. Weisbrod and coworkers reported that up to 29% of patients who suffered penetrating TBI in combat due to gunshots or blast had systemic infections, and 25% had meningitis during acute hospitalization; up to 39% developed epilepsy in a 2-year follow-up [21]. In another study, Saadat and coworkers found a poor outcome if the military perforating injury was associated with CNS infection. CNS infections co-occurred with epilepsy, but the association between epilepsy and infections was not specifically analyzed [22]. A recent study demonstrated that elevated systemic levels of lipopolysaccharide (LPS), a component of the outer membrane of Gram-negative bacteria, due to the breakdown of the gastrointestinal barrier was associated with PTE in a rat lateral fluid-percussion injury (FPI) model [23]. Despite the important clinical ramifications, little is known about whether or not the re-activation of inflammation by peripheral infection in an adult TBI-primed brain facilitates epileptogenesis.

The present study was designed to test the hypothesis that LPS-induced peripheral infection at a chronic time-point after TBI in rats will serve as a “second hit,” thereby increasing neuronal excitability in the perilesional cortex and hippocampus and facilitating post-traumatic epileptogenesis.

## 2. Materials and Methods

**Figure 1:**
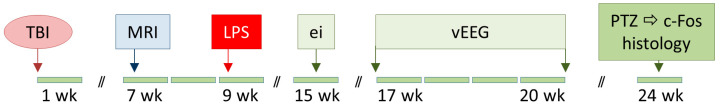
Study design. Traumatic brain injury (TBI) was induced by lateral fluid-percussion injury (FPI). Lesion endophenotype (focal inflammatory [TBI_FI_] vs. cavity-forming [TBI_CF_]) was assessed with T2-weighted magnetic resonance imaging (MRI) at 6 weeks after TBI. At 8 weeks post-TBI, rats received a single injection of lipopolysaccharide (LPS; 5 mg/kg, i.p.) or vehicle. Epidural skull electrodes (ei) were implanted at 14 weeks following TBI. Thereafter, 4-week-long video-electroencephalogram (vEEG) monitoring was performed starting at 16 weeks after TBI to detect spontaneous seizures. The pentylenetetrazole (PTZ) seizure-susceptibility test was performed under vEEG at 23 weeks post-TBI (i.e., 15 weeks after LPS injection). Finally, rats were perfused for histology at 2 h after PTZ administration (~6 months post-TBI). Sham-operated controls underwent all of the same procedures except the induction of TBI.

The study design is summarized in Figure 1.

### 2.1. Animals

A total of 46 adult (10-week-old) male Sprague–Dawley rats (300–350 g, Harlan Netherlands B.V., Udine, Italy) were used. Throughout the experiments, animals were housed in individual cages in a controlled environment (temperature 22 ± 1 °C; humidity 50 ± 10%; 12 h light/12 h dark cycle). Food and water were available ad libitum. All animal procedures were approved by the Animal Ethics Committee of the Provincial Government of Southern Finland and carried out in accordance with the European Council Directive (2010/63/EU).

### 2.2. Induction of TBI by Lateral FPI

Traumatic brain injury (TBI) was triggered by lateral FPI, as described previously [24]. Briefly, animals (*n* = 26) were anesthetized with a cocktail (6 mL/kg, i.p.) containing sodium pentobarbital (58 mg/kg), magnesium sulfate (127.2 mg/kg), propylene glycol (42.8%), and absolute ethanol (11.6%). A craniectomy (Ø 5 mm) was performed over the left parieto-temporal cortex between lambda and bregma (anterior edge 2.0 mm posterior to bregma; lateral edge adjacent to the left lateral ridge). The bone was carefully removed without disruption of the underlying dura. A female Luer-Lock connector was positioned into the craniotomy hole, its edges carefully sealed with Vetbond tissue adhesive (3M, St. Paul, MN, USA), and the cap filled with sterile saline and fixed to the skull with dental acrylate (Selectaplus powder #10009210; Selectaplus liquid CN #D10009102, DeguDent, Germany). About 90 min after administration of anesthesia cocktail, when the toe reflex reappeared, the rat was attached to the fluid-percussion device (AmScien Instruments, Richmond, VA, USA). Lateral FPI was induced by a transient (21–23 ms) fluid pulse impact against the exposed dura. The pendulum height was adjusted to produce severe injury [~3.0 atm; expected <72 h mortality 25%; [4]]. Sham-injured animals (*n* = 7) also underwent anesthesia and craniectomy procedures without exposure to lateral FPI. Naive animals (*n* = 5) were not subjected to anesthesia, craniectomy, or injury.

### 2.3. Magnetic Resonance Imaging (MRI)

Our previous MRI and histologic studies indicated inter-animal heterogeneity in the progression of cortical lesion pathology after lateral FPI-induced TBI [5,25,26,27]. To stratify the rats into different treatment groups based on the main lesion endophenotype [focal inflammatory (TBI_FI_) vs. cavity-forming (TBI_CF_)], rats underwent MRI at 6 weeks post-TBI. MRI was conducted under isoflurane anesthesia (1.5% isoflurane, O_2_/N_2_ 30/70% as carrier gas) at 9.4 T horizontal magnet (Varian Inc., Palo Alto, CA, USA) interfaced to Bruker Pharmascan console (Bruker Biospin, Ettlingen, Germany) using actively decoupled volume transmitter and quadrature surface receiver coils. Anatomical T2-weighted images were acquired using rapid acquisition with relaxation enhancement (RARE) sequence (TE 40 ms, TR 4000 ms, flip 90°, 25 slices, thickness 1 mm, a field of view 30 × 30 mm, 256 × 256 matrix, 2 averages, RARE factor 8) and T2 maps were acquired using multi-slice multi-echo (MSME) sequence (TR 5000 ms, TE 12, 24, 36, 48, 60, 72, 84, 96, 108, 120 ms, 10–15 slices to cover the lesion area, thickness 1 mm, interleaved collection, 256 × 128 matrix).

Anatomical T-weighted images were acquired at 7 T Bruker Pharmascan MRI scanner using fast spin-echo pulse sequence (TR 4000 ms, effective TE 40 ms, 25 slices, slice thickness 1 mm). In T2-weighted MRI, 10–15 slices that covered the entire rostrocaudal extent of the lesion were analyzed. Imaging was conducted using a 9.4 T horizontal magnet (Varian Inc., Palo Alto, CA, USA) interfaced to a Direct Drive console (Varian Inc.) as previously described by Immonen et al. [5]. According to the distribution and extent of signal intensity in T2-weighted MRI, 15 rats with TBI had developed the TBI_FI_ endophenotype and 11 had developed the TBI_CF_ endophenotype of cortical lesion by the time of the MRI.

### 2.4. Lipopolysaccharide (LPS) Injection

To model peripheral infection occurring during the post-TBI recovery phase caused by Gram-negative bacteria activating TLR4-mediated signaling [28], rats with the TBI_FI_ or TBI_CF_ endophenotype were randomized into the vehicle or LPS treatment groups. At 8 weeks post-TBI, animals received a single intraperitoneal injection of LPS (serotype 055:B5, Sigma-Aldrich; 5 mg/kg) or vehicle (0.9% NaCl, 2.5 mL/kg). Naïve and sham-operated animals were injected with vehicle only. This resulted in 6 different groups: Naïve-Veh (*n* = 5), Sham-Veh (*n* = 7), TBI_FI_-Veh (*n* = 7), TBI_FI_-LPS (*n* = 8), TBI_CF_-Veh (*n* = 5), TBI_CF_-LPS (*n* = 6).

### 2.5. Electrode Implantation for Electroencephalogram (EEG) Monitoring

To monitor the spontaneous and pentylenetetrazole (PTZ)-induced epileptiform activity, 4 stainless steel epidural screw electrodes, 1 reference electrode, and 1 ground electrode were inserted into the skull (Ø 1 mm, Plastics One, Inc., Roanoke, VA, USA) on weeks 15 after TBI as described by Kharatishvili et al. [29]. Video-EEG monitoring was initiated after a 7-d recovery period. A lost headset was re-implanted once if the skull was intact and not infected, and monitoring was continued.

### 2.6. Video-EEG Monitoring and Analysis of Occurrence of Spontaneous Seizures

A 4-weeks continuous (24/7) video-EEG (vEEG) monitoring was started on weeks 17 post-TBI to detect epileptiform activity as described in detail by [30].

Digital EEG files were scanned on the computer screen and manually analyzed by a blinded investigator. A spontaneous electroencephalographic seizure was defined as a high amplitude (more than twice baseline) rhythmic discharge that clearly represented a new pattern of tracing (repetitive spikes, spike-and-wave discharges, and slow waves) and lasted >5 s. Epileptic events occurring with an interval of less than 5 s without the EEG returning to baseline were defined as belonging to the same seizure. In addition, the occurrence of epileptiform discharges (EDs), defined as rhythmic transients (≥1 s, but <5 s) containing spikes and uniform sharp waves, was analyzed.

If an electrographic seizure was observed, its behavioral severity was assessed from the corresponding video recording according to a modified Racine’s scale [31]. As described previously by Rodgers et al. [32], we also noted the occurrence of spike-and-wave discharges in both the sham-operated and injured rats, but they were not counted as TBI-related seizures.

### 2.7. Pentylenetetrazole (PTZ) Seizure Susceptibility Test

Seizure susceptibility was assessed at 23 weeks post-TBI (i.e., 3 weeks after completing the continuous vEEG monitoring). Animals were placed in a transparent plexiglass cage (47 cm × 29 cm × 50 cm) and connected to the vEEG system 24 h before the test. After a baseline vEEG recording, animals were injected with a subconvulsive dose of PTZ (25 mg/kg, i.p., Sigma-Aldrich, YA-Kemia Oy, Finland) and continuously vEEG monitored for 120 min. As outcome parameters, we assessed (1) latency to the first spike (s), (2) latency to the first ED (s), (3) occurrence of electrographic seizures (yes/no), (4) latency to the first electrographic seizure (s), (5) duration of an electrographic seizure (s), and (6) number and severity of induced behavioral seizures [31].

### 2.8. Histology

*Processing of brain tissue.* At 120 min after PTZ injection, animals were disconnected from the vEEG, deeply anesthetized (as described before), and perfused intracardially with 4% paraformaldehyde in 0.1 M sodium phosphate buffer (PB), pH 7.4. Brains were post-fixed in 4% paraformaldehyde in 0.1 M PB, cryoprotected in 20% glycerol in 0.02 M potassium phosphate-buffered saline (KPBS, pH 7.4), frozen on dry ice, and stored at −70 °C until further processed.

*c-Fos immunohistochemistry.* A series of free-floating sections (1-in-10 series, 25 µm) was rinsed, and then, treated with 1% H_2_O_2_ in 0.02 M KPBS at room temperature (RT) for 15 min to remove endogenous peroxidase. Then, sections were incubated for 72 h at 4 °C in a primary antibody solution containing rabbit-polyclonal antibody raised against c-Fos (1:20 000, sc-253, Santa Cruz Biotechnology), 1% NGS, and 0.5% TX-100 in 0.02 M KPBS. After 3 washes in 2% NGS in 0.02 M KPBS, sections were incubated at RT for 1 h in a secondary antibody solution containing biotinylated goat anti-rabbit IgG (1:200, BA-1000, Vector Laboratories, Burlingame, CA, USA) with 1% NGS and 0.5% TX-100 in 0.02 M KPBS. After 3 washes, sections were incubated in avidin–biotin solution (1:200, PK-4000, Vector Laboratories) in 0.02 M KPBS for 45 min at RT. Then, sections were recycled into the secondary antibody solution (45 min at RT), washed 3 times, and recycled into avidin–biotin solution (30 min at RT). After 3 washes (0.02 M KPBS, 10 min each), the sections were incubated in a solution containing 0.1% 3′,3′-diaminobenzidine (DAB, Pierce Chemicals, Rockford, IL, USA) and 0.08 % H_2_O_2_ in 0.02 M KPBS for visualization of the staining. Then, the sections were mounted on gelatin-coated microscope slides, dried overnight at 37 °C and the reaction product was intensified with osmium (OsO_4_; #19170; Electron Microscopy Sciences, Hatfield, PA, USA) and thiocarbohydrate (TCH; #21900; Electron Microscopy Sciences) according to by Lewis et al. [33]. Finally, slides were covered using DePex as a mounting medium.

### 2.9. Assessment of the Density of c-Fos Immunoreactive Neurons in the Perilesional and Corresponding Contralateral Cortex

To assess the pattern of PTZ-induced neuronal activation in the brain undergoing epileptogenesis after TBI, we measured the distribution and density of c-Fos labeling in the cerebral cortex from digital photomicrographs of immunostained sections using Image J software (version 1.46r, http://rsb.info.nih.gov/ij/ accessed on 22 November 2021). For the analysis, we selected 2 sections from each rat: 1 from the most rostral and another from the most caudal level of the cortical lesion. A series of contiguous images was then captured from each section at 5× magnification, and a single montage image of the whole section was generated using a Zeiss Imager M2 microscope equipped with a Zeiss Axiocam 506 color camera operated by ZEN software. Four regions of interest (ROIs) were drawn in each section: a 1 mm wide cortical area bordering the lesion core medially (medial perilesional cortex) and laterally (lateral perilesional cortex), and the corresponding areas in the contralateral cortex. Each cortical area was further subdivided into the area containing layers II-IV and layers V-VI. This resulted in the following 4 ROIs: medial perilesional supragranular layers (including internal granular layer IV), medial perilesional infragranular layers, lateral perilesional supragranular layers, and lateral perilesional infragranular layers. Accordingly, the corresponding contralateral cortex was also divided into 4 areas, resulting in a total of 8 ROIs per section. Next, the RGB color images were converted into gray-scale images. Then, gray-scale images were thresholded manually to match with the c-Fos positivity in the immunostained section. Then, the (a) total area of the ROI (total number 8) and (b) area of c-Fos positivity in a thresholded image were calculated. The c-Fos expression-% (c-Fos-%) in each ROI was calculated as (c-Fos positive area/ROI area) × 100%.

### 2.10. Statistical Analysis

Statistical analysis was performed using SPSS for Windows (version 19.0) and GraphPad Prism5. The non-parametric Kruskal–Wallis test was used to assess differences in the parameters of the PTZ test (latency to the first electrographic seizure, latency to the first spike, latency to the first ED, seizure duration, and behavioral score) and the density of c-Fos expression between treatment groups. *Post hoc* analysis was performed using the Mann–Whitney U test. The Wilcoxon test was applied to test the differences in c-Fos expression between different brain areas (ipsilateral vs. contralateral, rostral vs. caudal, medial vs. lateral, supragranular layers vs. infragranular layers) within the same animal. The chi-square (χ²) test was applied to analyze differences in the occurrence of PTZ-induced seizures and increased c-Fos expression in the dentate gyrus between experimental groups. The non-parametric Spearman rank correlation test was used to analyze correlations between c-Fos expression density and the total seizure duration or the maximum behavioral score in the PTZ test. Data are presented as mean ± standard deviation or as mean ± standard error of the mean. Statistical significance was set at *p* < 0.05.

## 3. Results

### 3.1. Impact Pressure, Occurrence of Post-Impact Seizure-Like Behavior, Apnea Time, and Mortality

*Impact pressure.* The impact pressure used to induce lateral FPI was 3.23 ± 0.09 atm (range 2.91–3.39 atm). There was no difference between the rats that developed TBI_FI_ (3.21 ± 0.07) or TBI_CF_ (3.23 ± 0.11) endophenotypes at 6 weeks post-TBI (*p* = 0.096).

*Apnea.* The post-impact time in apnea was 20 ± 14 s (range 5–60 s). There was no difference between the TBI_FI_ (23 ± 16) and TBI_CF_ (17 ± 11) endophenotypes (*p* = 0.3709).

*Acute and follow-up mortality.* Acute post-impact mortality (<72 h) was 21% (7/33 rats with TBI), indicating moderate severity of the TBI [24,34,35]. Follow-up mortality (>72 h post-injury) was 31% (8/26) and typically occurred during the anesthesia-related to electrode implantation or unknown causes. In sham-operated animals, acute mortality was 0% (0/7) and follow-up mortality 28% (2/7; during electrode implantation-related anesthesia).

### 3.2. MRI Indicated Equal Distribution of TBI_FI_ and TBI_CF_ Endophenotypes at 6 Weeks Post-TBI

**Figure 2:**
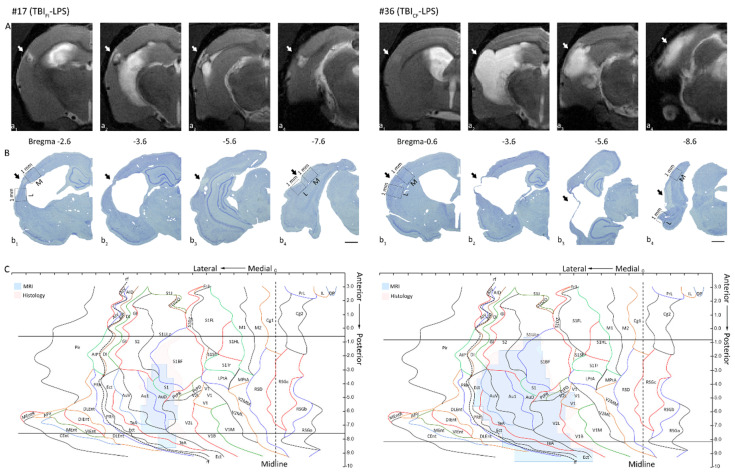
Cortical lesion endophenotypes. Representative unfolded MRI and histologic cortical maps of 2 rats with lateral fluid-percussion-induced traumatic brain injury (TBI), showing the extent and location of the cortical lesion in the focal-inflammatory (rat #17 from the TBI_FI_-LPS group; left panels) and cavity-forming (rat #36 from the TBI_CF_-LPS group; right panels) endophenotypes. The horizontal lines indicate the 2 rostrocaudal levels, from which the immunostained sections were sampled for analysis of c-Fos expression. (**A**) Four representative coronal T_2_-weighted MRI slices (a_1_ most rostral, a_4_ most caudal) used for the unfolding of the lesion in MRI images at 6 weeks post-TBI. White arrows indicate the lesion. (**B**) Four thionin-stained sections (23 weeks post-TBI) corresponding to levels of MRI slices in panel (**A**). Dashed 1 mm wide squares in b_1_ (rostral) and b_4_ (caudal), extending throughout layers I-VI indicate the areas used for the quantitative analysis of c-Fos-immunoreactivity (ir) in the medial (m) and lateral (l) perilesional cortex. (**C**) Unfolded MRI (blue) and histologic (pink) cortical lesion overlaid on the unfolded template prepared as previously described [26]. Abbreviations: CF, cavity-forming; FI, focal inflammatory; ir, immunoreactivity; L, lateral; LPS, lipopolysaccharide; M, medial; MRI, magnetic resonance imaging; TBI, traumatic brain injury. Scale bar = 1 mm.

Consistent with our previous follow-up studies [5,26,27], MRI analysis of cortical pathology at 6 weeks post-TBI (*n* = 26) indicated 2 major structural cortical lesion endophenotypes. One endophenotype was characterized by a focal cortical lesion surrounded by an enhanced T2 signal, reporting on ongoing perilesional inflammation, and is referred to here as “focal inflammatory endophenotype” (TBI_FI_) (Figure 2A). The second endophenotype presented as a large cortical cavity accompanied by an enlarged ipsilateral lateral ventricle and very narrow or no perilesional inflammation, referred to here as a “cavity-forming endophenotype” (TBI_CF_) (Figure 2B). At 6 weeks post-TBI, 58% (15/26) of the TBI rats had developed a TBI_FI_ endophenotype, and 42% (11/26) developed a TBI_CF_ endophenotype. Consequently, the 26 surviving rats of both endophenotypes were randomized into the LPS or vehicle treatment groups, resulting in 4 experimental groups: TBI_FI_-Veh (7), TBI_FI_-LPS (8), TBI_CF_-Veh (5), and TBI_CF_-LPS (6).

### 3.3. Spontaneous Seizures and Epileptiform Discharges

**Figure 3:**
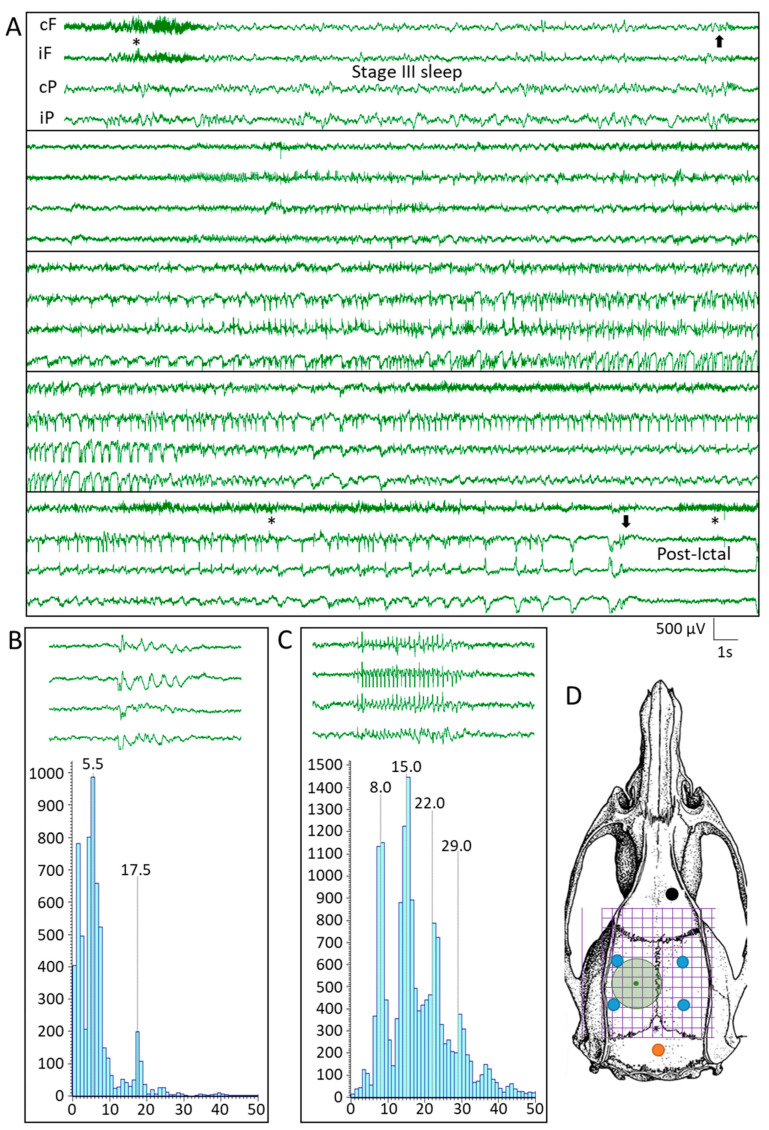
Video-electroencephalogram (vEEG) analysis. (**A**) A spontaneous seizure in a rat (#22) from the TBI_FI_-Veh group that occurred during the transition from stage III sleep to rapid eye movement sleep (REM). Black arrows indicate the beginning and end of the seizure. Asterisks refer to arousals. The duration of the electrographic seizure was 117 s, and the behavioral Racine score was 3 [31]. The X-Y scale in the right lower corner indicates the voltage and duration or electrographic patterns and applies to panels A-C. Stars indicate muscle artifacts. (**B**) An example of the epileptiform discharge (ED) lasting 1.2 s in a rat (#39) from the TBI_FI_-LPS group. Note the peak in relative power at 5.5 Hz. (**C**) An example of a spike-and-wave discharge (SWD) in a rat (#40) in the TBI_FI_-Veh group. Note the peak in relative power at 8 Hz and subsequent harmonics. SWDs were also found in the sham-operated group and were considered to be age-related oscillations in Sprague–Dawley rats]. (**D**) Green circle indicates the craniotomy. Positions of the 4 epidural recording electrodes (Ø 1 mm, blue circles), a reference electrode (black), and ground electrode (orange). Abbreviations: cF, contralateral frontal; cP, contralateral posterior; ED, epileptiform discharge; FI, focal inflammatory; iF, ipsilateral frontal; iP, ipsilateral posterior; LPS, lipopolysaccharide; REM, rapid eye movement sleep; SWD, spike-and-wave discharge; TBI, traumatic brain injury; Veh, vehicle.

In the 4-weeks vEEG monitoring that started on weeks 17 post-TBI (i.e., 8 weeks after LPS injection) 1 rat in the TBI_FI_-Veh group (#22) expressed 1 spontaneous seizure (Racine score 1) lasting for 117 s (Figure 3A). Another rat in the TBI_FI_-Veh group (#43) had a spontaneous seizure during the overnight vEEG recording preceding the PTZ test. The seizure lasted 185 s and had a Racine score of 5. In both animals, the seizures occurred during the transition from N3 sleep to REM (Figure 3A). No handling-related seizures were observed.

One of the 5 rats in the TBI_FI_-LPS group expressed EDs (Figure 3B, no spontaneous seizures were observed). All except 3 sham and TBI rats (1 in TBI_FI_-Veh, 1 in TBI_FI_-LPS, and 1 in TBI_CF_-LPS group) showed spike-and-wave discharges (SWDs, Figure 3C).

### 3.4. Peripheral Infection at 8 Weeks Post-TBI Increased Seizure Susceptibility in the PTZ Test

The effect of TBI with or without LPS treatment on seizure susceptibility was tested using the PTZ-test at 4 weeks after completing the 4-weeks vEEG monitoring, i.e., 23 weeks after TBI and 15 weeks after LPS injection. Data are summarized in Table 1.

*Occurrence of PTZ-induced seizures.* PTZ-induced seizures occurred more often in the TBI-LPS group than in the sham-injured group (80% vs. 20%, *p* < 0.05, χ^2^-test). Seizure occurrence did not differ between the TBI-LPS and TBI-Veh groups (80% vs. 46%, *p* > 0.05, χ^2^-test) (Table 1).

**Figure 4:**
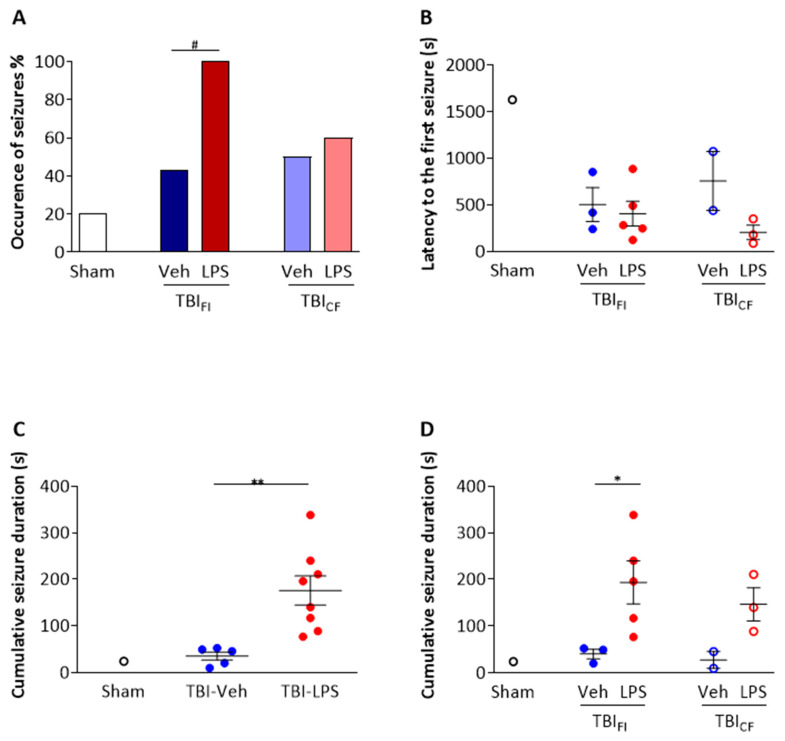
Effect of lesion endophenotype on PTZ seizure susceptibility test. (**A**) Occurrence of PTZ-induced seizures was increased by the “second hit” in the TBI_FI_ endophenotype (TBI_FI_-LPS 100% vs. TBI_FI_-Veh 43%). (**B**) Latency to the first electrographic seizure was not affected by the endophenotype of the cortical lesion. Please note that only a subpopulation of animals developed PTZ-induced seizures (e.g., only 1 rat showed a seizure after PTZ administration in the sham-operated group). (**C**) TBI-LPS rats showed a longer cumulative seizure duration (163 ± 90 s, range 77–338 s, median 168 s) compared with TBI-Veh rats (35 ± 19 s, range 10–52 s, median 46 s). (**D**) Further analysis revealed that the difference resulted from the prolonged cumulative seizure duration in the TBI_FI_ endophenotype. Data are presented as mean ± standard error of the mean (SEM) (panels (**B**,**C**)). Statistical significances: # *p* < 0.05 (χ^2^-test); * *p* < 0.05, ** *p* < 0.01 (Mann–Whitney U test). Abbreviations: CF, cavity-forming; FI, focal inflammatory; LPS, lipopolysaccharide; PTZ, pentylenetetrazole; TBI, traumatic brain injury; Veh, vehicle.

When the 2 endophenotypes were analyzed separately, PTZ-induced seizures within 1 h after PTZ injection occurred more often in the TBI_FI_-LPS group than in the TBI_FI_-Veh group (100% vs. 43%, *p* < 0.05, χ^2^-test). Occurrence of induced seizures in the TBI_CF_-LPS group, however, did not differ from that in the TBI_CF_-Veh group (60% vs. 50%, *p* > 0.05, χ^2^-test). In addition, there was no difference between the TBI_FI_-LPS and TBI_CF_-LPS groups (100% vs. 60%) or between the TBI_FI_-Veh and TBI_CF_-Veh groups (43% vs. 50%) (Figure 4A).

*Number of PTZ-induced seizures.* The mean number of PTZ-induced seizures did not differ between the TBI-LPS and TBI-Veh groups (1.8 ± 1.0 vs. 1 ± 0, *p* > 0.05). In addition, no differences were detected between the endophenotypes (data not shown).

*Latency to the first electrographic seizure.* Only 1 of the sham-operated rats developed a seizure after PTZ injection (latency 1 628 s). The latency to the first electrographic seizure in the TBI-LPS group tended to be shorter than that in the TBI-Veh group (331 ± 258 s vs. 604 ± 345 s, *p* > 0.05) (Table 1). Even though the different pathologic endophenotypes did not differ from each other, the latency to the first PTZ-induced seizures seemed the shortest in the TBI_CF_-LPS group (206 ± 133 s) (Figure 4B).

*Cumulative duration of PTZ-induced electrographic seizures.* Cumulative seizure duration was longer in TBI-LPS rats (163 ± 90 s, range 77–338 s, median 168 s) than in TBI-Veh rats (35 ± 19 s, range 10–52 s, median 46 s, *p* < 0.01) (Figure 4C). In particular, the cumulative seizure duration in the TBI_FI_-LPS group (193 ± 103 s, range 77–338 s, median 196 s) was prolonged compared with that in the TBI_FI_-Veh group (40 ± 18 s, range 20–52 s, median 49 s, *p* < 0.05) (Figure 4D). It should be noted that rats in the TBI_CF_-LPS group also tended to have a prolonged cumulative seizure duration compared with the TBI_CF_-Veh group. As only 2 rats in the TBI_CF_-Veh group had PTZ-induced seizures, however, the difference was not significant (Figure 4D).

*Behavioral severity of PTZ-induced seizures.* The mean seizure behavioral score was 4.0 ± 1.0 (range 3–5, median 4) in the TBI-LPS group, 3.2 ± 2.0 (range 0–5, median 3) in the TBI-Veh group, and 3 in the sham group (only 1 seizure) (Table 1). No differences were detected between groups or endophenotypes (data not shown).

*Latency to the first spike.* The latency to the first spike after PTZ treatment tended to be reduced in the TBI-LPS group compared with the TBI-Veh group (Table 1). The difference did not reach statistical significance, however, regardless of whether the 2 endophenotypes were analyzed together or separately (data not shown).

*Latency to the first ED.* Similarly, the latency to the first ED after PTZ administration tended to be shorter in the TBI-LPS group compared with the TBI-Veh group (Table 1). The difference did not reach statistical significance, however, regardless of whether the 2 endophenotypes were analyzed together or separately (data not shown).

### 3.5. Peripheral Infection at 8 Weeks Post-TBI Enhanced PTZ-Induced c-Fos Expression in the Perilesional Cortex and Dentate Gyrus

We focused our analysis of c-Fos activation on the perilesional cortex and hippocampus, which are known to be involved in PTE-related excitability in the lateral FPI model [27,29,36]. Our initial visual analysis of immunostained preparations revealed differences in PTZ-induced nuclear c-Fos expression (a) along with the rostrocaudal extent of the cortical lesion, (b) between the supragranular and infragranular cortical layers, (c) between the TBI_FI_ and TBI_CF_ endophenotypes (both cortical and hippocampal c-Fos expression), (d) between LPS- and vehicle-treated injured animals, and (e) between rats with or without induced seizures in the PTZ test.

### 3.6. Topography of PTZ-Induced c-Fos Expression along the Rostrocaudal Extent of Cortical Lesion

**Figure 5:**
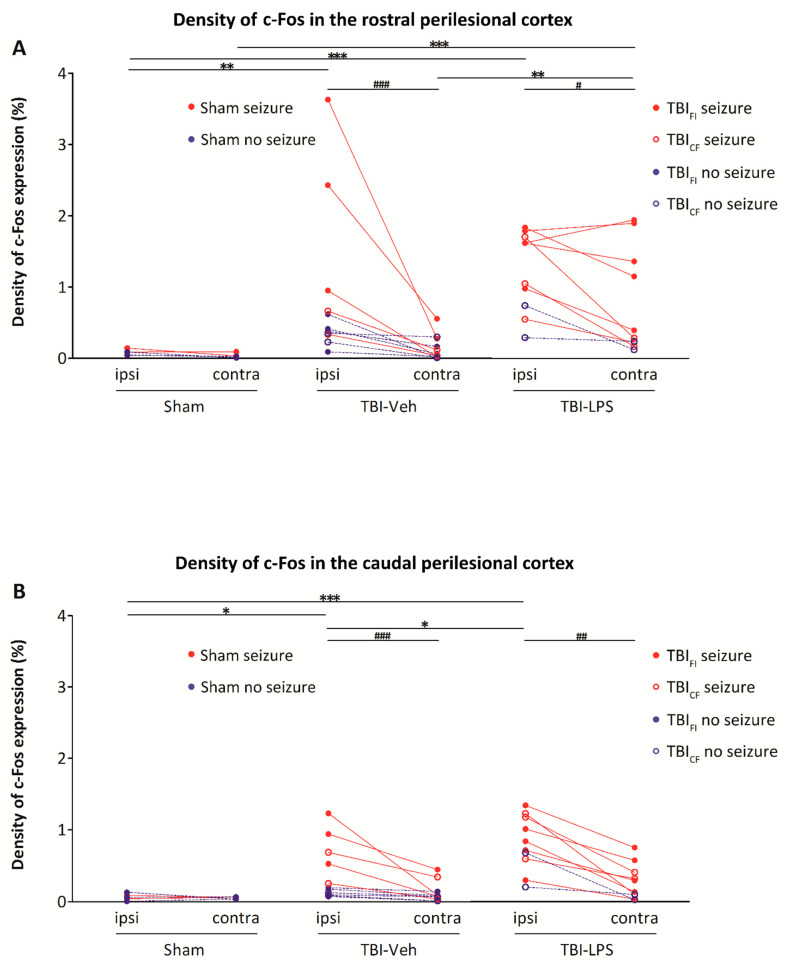
c-Fos expression in the rostral and caudal perilesional cortex. (**A**) *Rostral perilesional cortex.* In the Sham-Veh group, the density of c-Fos expression was comparable between the ipsilateral and contralateral perilesional cortex rostrally. In the TBI-Veh group, c-Fos expression was increased ipsilaterally compared with the Sham-Veh group. In the TBI-LPS group, the density of c-Fos expression was higher both ipsilaterally and contralaterally than in the Sham-Veh group. Contralateral labeling was also higher than that in the TBI-Veh group. Inter-hemispheric analysis showed higher c-Fos labeling ipsilaterally than contralaterally in both the TBI-Veh and TBI-LPS groups. (**B**) *Caudal perilesional cortex*. In the Sham-Veh group, the density of c-Fos expression was comparable between the ipsilateral and contralateral perilesional cortex caudally. The c-Fos labeling density was higher ipsilaterally in the TBI-Veh group than in the Sham-Veh group. In the TBI-LPS group, c-Fos expression was increased ipsilaterally compared with the Sham-Veh and TBI-Veh groups. Interhemispheric analysis revealed more c-Fos activation ipsilaterally than contralaterally in both the TBI-Veh and TBI-LPS groups. Comparison of c-Fos expression between the rostral and caudal perilesional cortex showed that c-Fos expression in the ipsilateral perilesional cortex was higher rostrally than caudally in the TBI-Veh group (*p* < 0.05, Wilcoxon). In the TBI-LPS group, rostral c-Fos expression was increased bilaterally compared with the caudal c-Fos expression (*p* < 0.05, Wilcoxon). Abbreviations: CF, cavity-forming; contra, contralateral; FI, focal inflammatory; ipsi, ipsilateral; LPS, lipopolysaccharide; TBI, traumatic brain injury; Veh, vehicle. Statistical significances: # *p* < 0.05, ## *p* < 0.01, ### *p* < 0.001 (Wilcoxon); * *p* < 0.05, ** *p* < 0.01, *** *p* < 0.001 (Mann–Whitney *U* test).

To assess the topography of PTZ-induced c-Fos activation, we divided the lesioned cortex into the medial and lateral perilesional cortex, which were analyzed both rostrally and caudally. As shown in detail below, the c-Fos activation in the TBI-Veh group was greater in the lateral perilesional cortex (area closer to the rhinal fissure) compared with the medial perilesional cortex (closer to midline; *p* < 0.05, Supplementary Figures S1–S4). In addition, the rostral areas were more activated than the caudal areas after TBI (Figure 5 and significances therein). In sham-operated animals, no medial-lateral or rostral-caudal gradients were observed.

*Rostral perilesional cortex.* In the Sham-Veh group, the density of c-Fos expression was comparable between the ipsilateral (side of craniotomy) and contralateral rostral cortex (AP from −0.96 to −1.80 from the bregma; the levels correspond to rostral levels of the cortical lesion in TBI rats).

Ipsilateral perilesional c-Fos expression was greater in the TBI-Veh group than in the Sham-Veh group (*p* < 0.01). Ipsilateral labeling was also higher than that contralaterally (*p* < 0.001) (Figure 5A).

In the TBI-LPS group, c-Fos expression was increased both ipsilaterally and contralaterally compared with the Sham-Veh group (both *p* < 0.001). Contralateral labeling was also higher than that in the TBI-Veh group (*p* < 0.01). Such as in the TBI-Veh group, the labeling was higher ipsilaterally than contralaterally. (*p* < 0.05) (Figure 5A).

*Caudal perilesional cortex.* In the Sham-Veh group, the density of c-Fos expression was comparable between the ipsilateral and contralateral caudal cortex (AP −5.88 to −6.24 from bregma; the levels correspond to caudal levels of the cortical lesion in TBI rats).

The density of ipsilateral c-Fos labeling was greater in the TBI-Veh group than in the Sham-Veh group (*p* < 0.05). Ipsilateral labeling was also higher than that contralaterally (*p* < 0.001) (Figure 5B).

Ipsilateral c-Fos expression was greater in the TBI-LPS group than in the Sham-Veh group (*p* < 0.001). Ipsilateral labeling was also higher than that in the TBI-Veh group (*p* < 0.05). Such as in the TBI-Veh group, the labeling was higher ipsilaterally than contralaterally (*p* < 0.01) (Figure 5B).

*Rostral vs. caudal perilesional cortex.* In the TBI-Veh group, ipsilateral perilesional c-Fos expression was greater rostrally than caudally (*p* < 0.05). In the TBI-LPS group, both ipsilateral and contralateral c-Fos expression was greater rostrally than caudally (both *p* < 0.05) (Figure 5).

### 3.7. Laminar Analysis of PTZ-Induced c-Fos Expression

Next, we assessed c-Fos expression in layers II-IV (supragranular layers) and layers V-VI (infragranular layers) in the rostral perilesional cortex, in which we found the highest c-Fos expression levels. As shown in Supplementary Figure S2, most of the c-Fos expression was in layers II-IV (medial and lateral perilesional cortex combined). The levels were highest in animals that expressed seizures in the PTZ-test, whether or not they had been treated with vehicle or LPS. Compared with the contralateral side, there was a clear asymmetry in the TBI-Veh group (*p* < 0.001). In the TBI-LPS group, however, c-Fos expression in the superficial layers was increased bilaterally.

In addition, in the caudal perilesional cortex, the most robust activation was observed in layers II-IV (data not shown).

### 3.8. Effect of Lesion Endophenotype on the Pattern of c-Fos Expression

**Figure 6:**
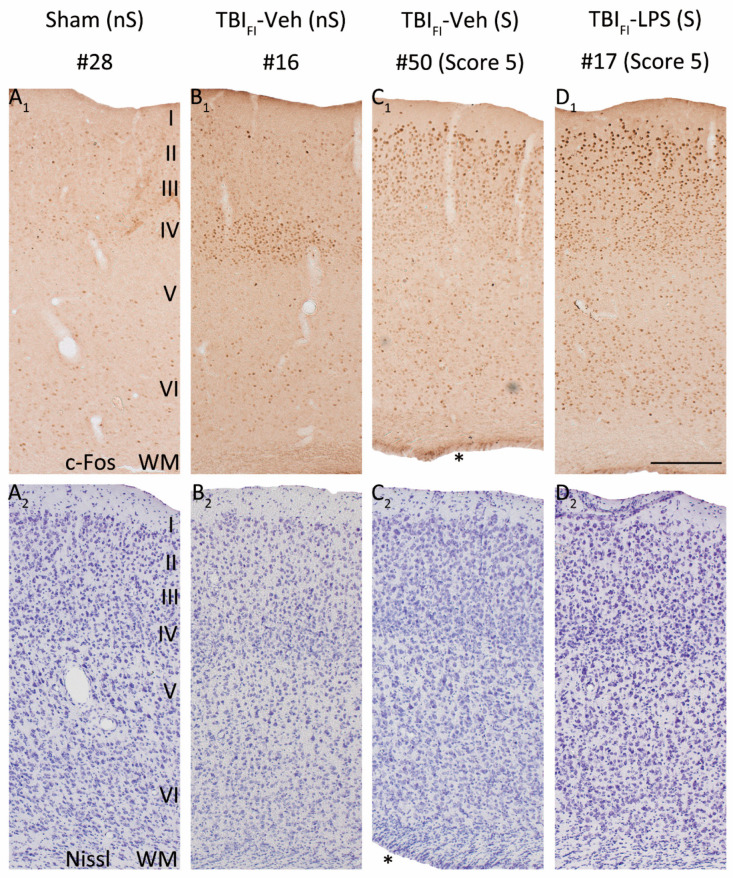
LPS enhances the seizure-induced c-Fos expression and changes its pattern in the rostromedial perilesional cortex. Representative high-magnification photomicrographs showing c-Fos immunolabeling (**A_1_**–**D_1_**) and thionin staining (**A_2_**–**D_2_**) in the rostromedial perilesional cortex (somatosensory cortex, see Figure 3C) of a vehicle-treated sham-operated rat and a vehicle or LPS-treated injured rat with a TBI_FI_ endophenotype at 2 h after PTZ injection (15 weeks after LPS injection and 23 weeks post-TBI). (**A_1_**,**A_2_**) A rat from the Sham-Veh group without PTZ-induced seizure (#28). Note the very low c-Fos expression level throughout the cortical layers. (**B_1_**,**B_2_**) A rat from the TBI_FI_-Veh group without a PTZ-induced seizure (#16). Note the intense c-Fos labeling in layer IV apparently reporting on the TBI-induced chronic excitability. (**C_1_**,**C_2_**) A rat from the TBI_FI_-Veh group with a PTZ-induced seizure (#50, Racine score 5 seizure). Note the intense c-Fos labeling in layers II-III. (**D_1_**,**D_2_**) A rat from the TBI_FI_-LPS group with a PTZ-induced seizure (#17, Racine score 5 seizure) with intense c-Fos labeling in layers II-III and scattered immunopositive cells in deeper layers. Note that the occurrence of PTZ-induced seizures changed the overall pattern of perilesional c-Fos expression in TBI animals (with or without LPS). In addition to labeling in layer IV, layers I-III were activated (e.g., (**D_1_**) vs. (**B_1_**)). A rat in the TBI_FI_-Veh group with PTZ-induced seizure (**C_1_**) showed higher c-Fos activation than a rat without a seizure ((**C_1_**) vs. (**B_1_**)), particularly in the supragranular layers. An LPS-treated rat with a TBI_FI_ endophenotype and a PTZ-induced seizure had robustly enhanced c-Fos expression in all cortical layers (**D_1_**) compared with a vehicle-treated seizing TBI rat (**C_1_**). Abbreviations: FI, focal inflammatory; FPI, fluid-percussion injury; LPS, lipopolysaccharide; nS, no seizure; PTZ, pentylenetetrazole; S, seizure; TBI, traumatic brain injury; Veh, vehicle; WM, white matter. Scale bars = 200 μm. * Enlarged ipsilateral lateral ventricle.

**Figure 7:**
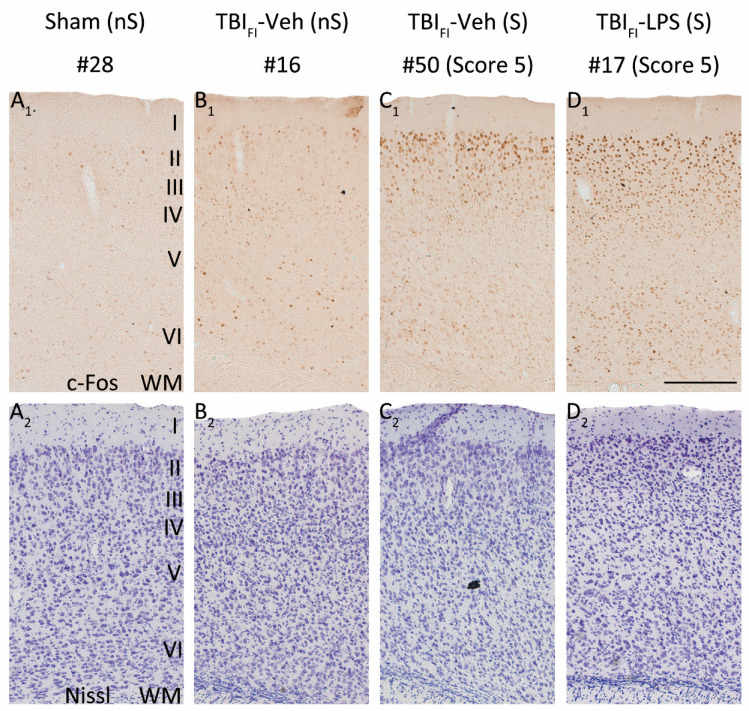
LPS enhances the seizure-induced c-Fos expression and changes its pattern in the caudomedial perilesional cortex. Representative high magnification photomicrographs showing c-Fos immunochemistry (**A_1_**–**D_1_**) and Nissl staining (**A_2_**–**D_2_**) in the caudomedial perilesional cortex in rats from the Sham-Veh and TBI_FI_ endophenotype groups at 2 h after PTZ injection (23 weeks post-FPI and 15 weeks after LPS injection). (**A_1_**,**A_2_**) Example of a rat from the Sham-Veh group without PTZ-induced seizure (#28). (**B_1_**,**B_2_**) Example of a rat from the TBI_FI_-Veh group without a PTZ-induced seizure (#16). (**C_1_**,**C_2_**) Example of a rat from TBI_FI_-Veh group with a PTZ-induced seizure (#50, seizure Racine score 5). (**D_1_**,**D_2_**) Example of a rat from TBI_FI_-LPS group with a PTZ-induced seizure (#17, seizure Racine score 5). Note that in rats without a PTZ-induced seizure, TBI increased PTZ-induced c-Fos expression ((**B_1_**) vs. (**A_1_**)). In the TBI_FI_-Veh group, a rat with a PTZ-induced seizure (**C_1_**) exhibited higher c-Fos activation compared to a rat without a seizure (**B_1_**), particularly in the supragranular layers. In the TBI_FI_ endophenotype with PTZ-induced seizure, LPS treatment at a chronic time-point after TBI further enhanced c-Fos expression (**D_1_**) compared with vehicle treatment (**C_1_**) in all layers. Abbreviations: FI, focal inflammatory; FPI, fluid-percussion injury; LPS, lipopolysaccharide; nS, no seizure; PTZ, pentylenetetrazole; S, seizure; TBI, traumatic brain injury; Veh, vehicle; WM, white matter. Scale bars = 200 μm.

**Figure 8:**
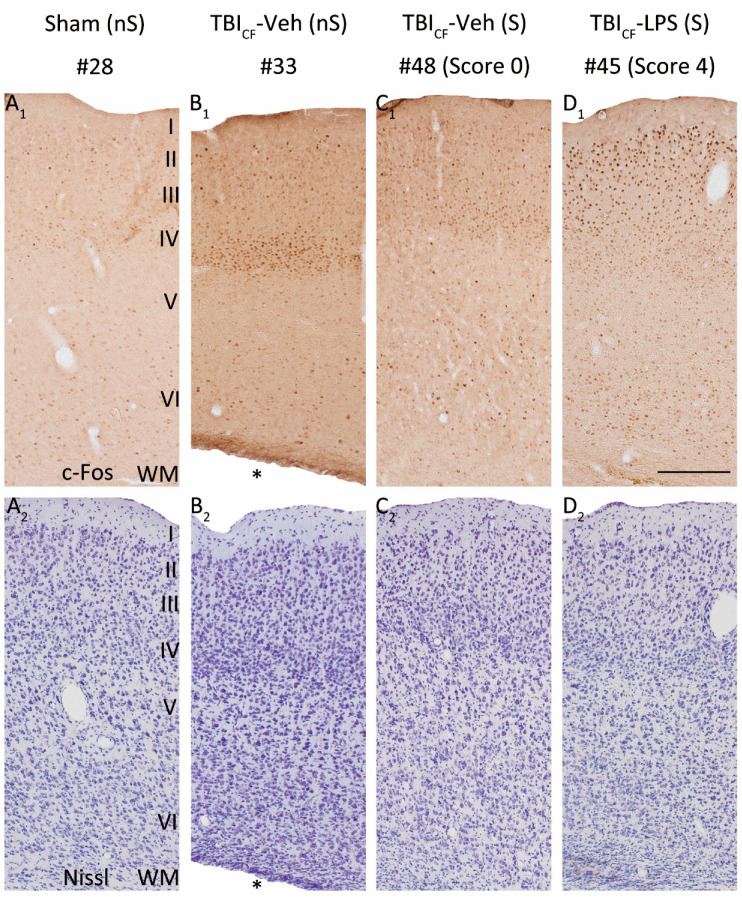
Pattern of c-Fos expression in the rostromedial perilesional cortex. Representative high magnification photomicrographs showing c-Fos immunochemistry (**A_1_**–**D_1_**) and Nissl staining (**A_2_**–**D_2_**) in the rostromedial perilesional cortex in rats from the Sham-Veh group and TBI_CF_ endophenotype at 2 h after PTZ injection (23 weeks post-FPI and 15 weeks after LPS injection). (**A_1_**,**A_2_**) Example of a rat from the Sham-Veh group without a PTZ-induced seizure (#28). (**B_1_**,**B_2_**) Example of a rat from the TBI_CF_-Veh group without a PTZ-induced seizure (#33). (**C_1_**,**C_2_**) Example of a rat from the TBI_CF_-Veh group with a PTZ-induced seizure (#48, seizure Racine score 0, only electroencephalographic seizure). (**D_1_**,**D_2_**) Example of a rat from the TBI_CF_-LPS group with a PTZ-induced seizure (#45, seizure Racine score 4). Note that in rats without a PTZ-induced seizure, TBI increased PTZ-induced c-Fos expression, particularly in layer IV ((**B_1_**) vs. (**A_1_**)). In the TBI_CF_-Veh group, a rat with a PTZ-induced seizure (**C_1_**) revealed higher c-Fos activation compared to a rat without a seizure (**B_1_**), particularly in the supragranular layers. In the TBI_CF_ endophenotype with a PTZ-induced seizure, LPS treatment at a chronic time-point after TBI further enhanced c-Fos expression (**D_1_**) compared with vehicle treatment (**C_1_**) in all layers. Abbreviations: CF, cavity-forming; FPI, fluid-percussion injury; LPS, lipopolysaccharide; nS, no seizure; PTZ, pentylenetetrazole; S, seizure; TBI, traumatic brain injury; Veh, vehicle; WM, white matter. Scale bars = 200 μm. * Enlarged ipsilateral lateral ventricle.

**Figure 9:**
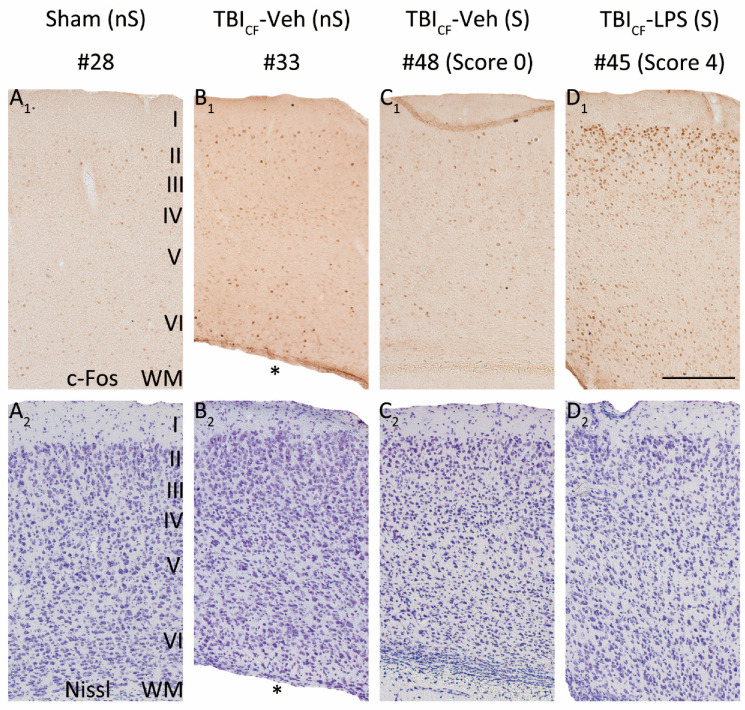
Pattern of c-Fos expression in the caudomedial perilesional cortex. Representative high magnification photomicrographs showing c-Fos immunochemistry (**A_1_**–**D_1_**) and Nissl staining (**A_2_**–**D_2_**) in the caudomedial perilesional cortex in rats from Sham-Veh group and TBI_CF_ endophenotype at 2 h after PTZ injection (23 weeks post-FPI and 15 weeks after LPS injection). (**A_1_**,**A_2_**) Example of a rat from the Sham-Veh group without a PTZ-induced seizure (#28). (**B_1_**,**B_2_**) Example of a rat from the TBI_CF_-Veh group without a PTZ-induced seizure (#33). (**C_1_**,**C_2_**) Example of a rat from the TBI_CF_-Veh group with a PTZ-induced seizure (#48, seizure Racine score 0, only electroencephalographic seizure). (**D_1_**,**D_2_**) Example of a rat from the TBI_CF_-LPS group with a PTZ-induced seizure (#45, seizure Racine score 4). Note that in rats without a PTZ-induced seizure, TBI increased PTZ-induced c-Fos expression ((**B_1_**) vs. (**A_1_**)). In the TBI_CF_-Veh group, no difference was detected between rats with a PTZ-induced seizure (**C_1_**) and those without a seizure (**B_1_**). In the TBI_CF_ endophenotype with a PTZ-induced seizure, LPS treatment at a chronic time-point after TBI further enhanced c-Fos expression (**D_1_**) compared with vehicle treatment (**C_1_**) in all layers. Abbreviations: CF, cavity-forming; FPI, fluid-percussion injury; LPS, lipopolysaccharide; nS, no seizure; PTZ, pentylenetetrazole; S, seizure; TBI, traumatic brain injury; Veh, vehicle; WM, white matter. Scale bars = 200 μm. * Enlarged ipsilateral lateral ventricle.

As the cumulative duration of PTZ-induced seizures was longer in the TBI_FI_ rats than in the TBI_CF_ rats, the highest activation of c-Fos expression was observed in rats with the post-TBI_FI_ endophenotype, whether or not they were treated with LPS (Figure 5, red lines with closed circles). Figure 6 and Figure 7 show representative photomicrographs PTZ-induced c-Fos activation of the rat with TBI_FI_ and Figure 8 and Figure 9 with TBI_CF_ endophenotype.

### 3.9. Effect of the Occurrence of PTZ-Induced Seizures on the Pattern of c-Fos Expression

*Perilesional cortex.* As only a subgroup of animals expressed electrographic seizures after PTZ administration, we next compared the activation patterns between animals with or without PTZ-induced seizures.

As summarized in Figure 5, in both the TBI-Veh and TBI-LPS groups, the highest densities of c-Fos activation were observed in rats with PTZ-induced seizures both rostrally and caudally (Figure 5, red circles and lines). Moreover, the activation was more robust ipsilaterally than contralaterally except in the TBI-LPS group, in which the rostral activation was high bilaterally (Figure 5 and significances therein). It should be noted that the injured rats without PTZ-induced seizures also tended to have higher c-Fos expression ipsilaterally than contralaterally (Figure 5).

**Figure 10:**
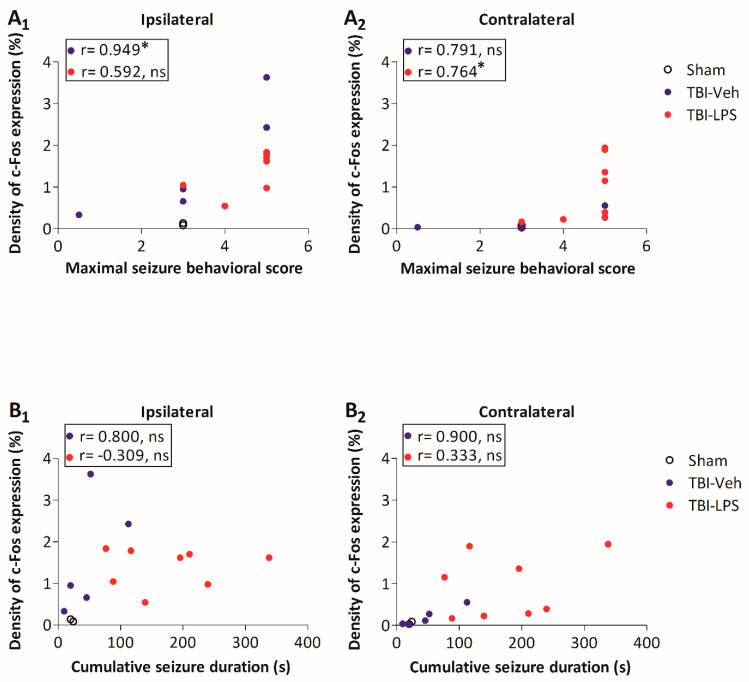
c-Fos expression and seizure susceptibility. Correlations between the density of rostral perilesional c-Fos expression and seizure susceptibility (maximal behavioral seizure score, cumulative seizure duration) in the PTZ-test. (**A_1_**) Ipsilaterally, the higher the density of c-Fos expression, the higher the maximal behavioral seizure score in the TBI-Veh group (r = 0.949, *p* < 0.05). (**A_2_**) Contralaterally, the higher the c-Fos expression, the higher the maximal behavioral score in the TBI-LPS group (r = 0.764, *p* < 0.05). No correlations between the density of c-Fos expression and the cumulative seizure duration were detected (**B_1_**) ipsilaterally or (**B_2_**) contralaterally in the TBI-Veh or TBI-LPS groups. Abbreviations: LPS, lipopolysaccharide; ns, non-significant; r, correlation coefficient; TBI, traumatic brain injury; Veh, vehicle. Statistical significances: * *p* < 0.05 (r, Spearman’s rho correlations).

Next, we assessed whether the density of c-Fos expression was associated with the maximal behavioral seizure score. In the TBI-Veh group, the higher the behavioral score, the greater the c-Fos expression in the ipsilateral rostral perilesional cortex (r = 0.9487, *p* < 0.05) (Figure 10). In the TBI-LPS group, the higher the behavioral seizure score, the higher the c-Fos expression in the contralateral cortex (r = 0.7638, *p* < 0.05) (Figure 10). No associations were detected between the cumulative seizure duration and c-Fos activation in the TBI-Veh and TBI-LPS groups (Figure 10).

*Dentate gyrus.* Szyndler et al. [37] reported increased c-Fos immunoreactivity in the dentate gyrus in rats that showed a stage 5 generalized tonic-clonic seizure after PTZ induction (35 mg/kg, i.p., repeated administration). Due to the increased seizure susceptibility of TBI rats to PTZ-induced seizures, we administered PTZ at a dose of 25 mg/kg. Consequently, none of the sham-operated animals developed stage 4–5 behavioral seizures, and none of them showed c-Fos activation in the dentate gyrus.

In the TBI-Veh group, 9 of 11 rats had a behavioral seizure score < 4 or no seizure in the PTZ-test. Interestingly, 1 of the rats showed increased c-Fos activation in the dentate gyrus. This rat expressed a spontaneous seizure lasting 185 s approximately 23 h before the PTZ injection (in baseline vEEG). Of the 11 rats in the TBI-Veh group, 2 scored 4–5 seizures after PTZ administration, and both of these rats had increased c-Fos activation in the dentate gyrus.

**Figure 11:**
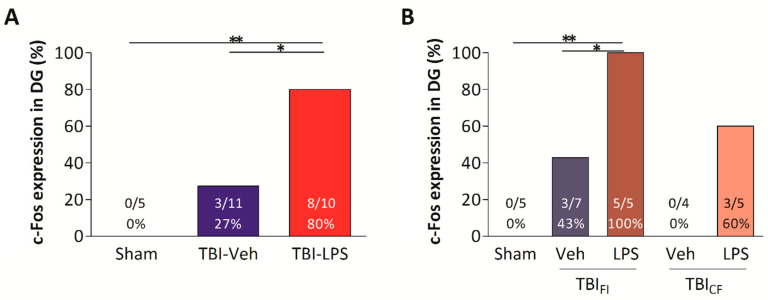
c-Fos expression in the dentate gyrus (DG). (**A**) Percentage of animals with increased c-Fos expression in the dentate gyrus was higher in the TBI-LPS than in the TBI-Veh and Sham-Veh groups. (**B**) All rats in the TBI_FI_-LPS group more commonly showed increased c-Fos activation in the dentate gyrus than rats in the TBI_FI_-Veh and Sham-Veh groups. Abbreviations: CF, cavity-forming; DG, dentate gyrus; FI, focal inflammatory; LPS, lipopolysaccharide; TBI, traumatic brain injury; Veh, vehicle. Statistical significances: * *p* < 0.05, ** *p* < 0.01 (χ^2^-test).

In the TBI-LPS group, 3 of 10 animals had a behavioral seizure score < 4 or no seizure in the PTZ-test. One of these rats (seizure score 3) showed increased c-Fos activation in the dentate gyrus. Of the 10 rats, 7 developed seizures that reached stage 4–5 within 2 h after PTZ administration, and all of these rats had increased c-Fos activation in the dentate gyrus. Consequently, the c-Fos expression increase in the dentate gyrus was higher in the TBI-LPS group (8/10 rats) than in the TBI-Veh group (3/11 rats, *p* < 0.05, χ^2^-test) or the sham group (0/5 rats, *p* < 0.01, χ^2^-test) (Figure 11).

**Figure 12:**
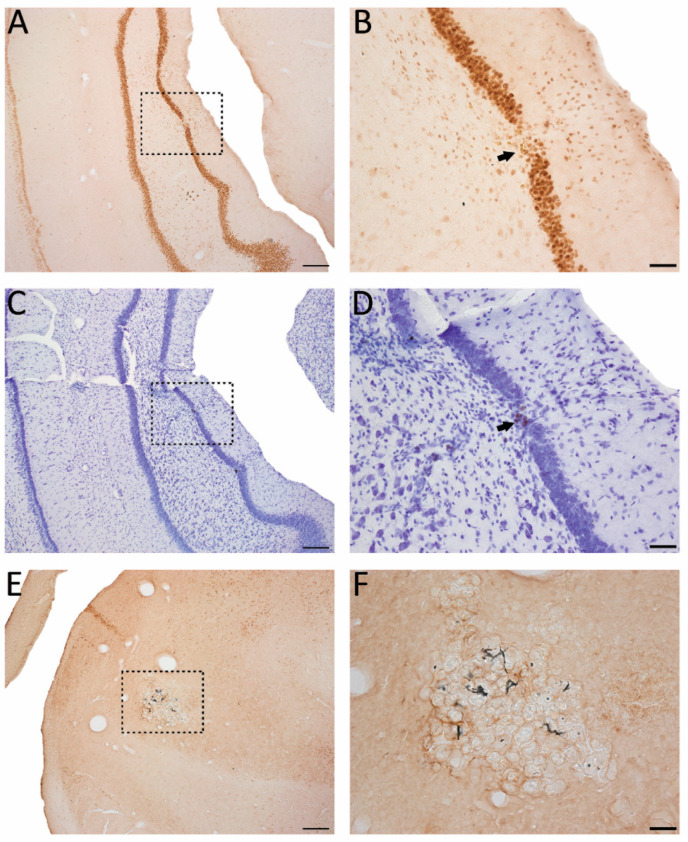
Pattern of c-Fos expression in the dentate gyrus. Representative photomicrographs showing mild granule cell damage and dispersion in the dentate gyrus (**A**–**D**) and calcifications in the ipsilateral thalamus (**E**,**F**) in a rat (#38, Racine score 5) in the TBI_FI_-Veh group with an induced seizure at 2 h after PTZ injection. A c-Fos immunostained (**A**) and a Nissl-stained section (**C**). A higher-magnification photomicrographs of c-Fos immunohistochemistry (**B**) and Nissl staining (**D**) taken from the region indicated with a dashed box in panel (**A**,**C**), respectively. Note the granule cell loss indicated by a closed arrow with iron deposits (dark dots) and dispersed granule cells in the molecular layer. (**E**) Thalamic calcification (dashed box). (**F**) A higher magnification photomicrograph was taken from the region indicated with a dashed box in panel (**E**). Abbreviations: FI, focal inflammatory; FPI, fluid-percussion injury; PTZ, pentylenetetrazole; TBI, traumatic brain injury; Veh, vehicle. Scale bar = 200 μm (panel (**A**,**C**,**E**)); 50 μm (panel (**B**,**D**,**F**)).

In the TBI_FI_ endophenotype, activation of the dentate gyrus was more common in the TBI_FI_ -LPS group than the TBI_FI_ -Veh group (100% vs. 43%, *p* < 0.05, χ^2^-test) or the sham group (100% vs. 0%, *p* < 0.01, χ^2^-test) (Figure 12). In the TBI_CF_ endophenotype, c-Fos activation in the dentate gyrus did not differ between the vehicle- and LPS-treated animals (0% vs. 60%, *p* > 0.05) (Figure 11).

In all animals, the increase in c-Fos expression was bilateral along the septotemporal axis of the dentate gyrus.

## 4. Discussion

The aim of the present study was to identify factors that facilitate epileptogenesis after TBI. We hypothesized that LPS treatment at 8 weeks after TBI, mimicking Gram-negative peripheral infection at a chronic post-injury time-point, will increase neuronal excitability and facilitate post-traumatic epileptogenesis. We had three major findings. First, LPS injection at 8 weeks post-TBI increased seizure susceptibility, particularly in rats with the TBI_FI_ endophenotype. Second, LPS augmented PTZ-induced c-Fos expression, a marker of neuronal activation in the injured ipsilateral cortex. Third, LPS enhanced PTZ-induced c-Fos bilateral expression in the dentate gyrus, particularly in the TBI_FI_ endophenotype.

### 4.1. Occurrence of Late Spontaneous Seizures

Previous studies demonstrated that approximately 10% of rats with lateral FPI have epilepsy at 3 months, 25% at 6–7 months, and 40% to 50% at 12 months post-injury [29]. Consistent with previous studies, approximately 10% of rats with TBI had electrographic seizures when vEEG-monitored during the fifth post-injury month. Consequently, we used PTZ-induced seizure susceptibility rather than the occurrence of spontaneous seizures as an outcome measure when assessing epileptogenesis in different treatment groups.

### 4.2. Peripheral Infection at a Chronic Time-Point Post-TBI Increased Seizure Susceptibility

Several studies revealed that peripheral inflammation in normal immature and/or mature rodents induced by LPS increases seizure susceptibility to convulsants, hyperthermic exposure, or kindling [38,39,40,41,42,43,44]. Our 6-month follow-up study extended previous observations by showing that LPS injection at 2 months after TBI, modeling peripheral Gram-negative infection in subjects with brain injury, enhanced seizure susceptibility in the PTZ test. This is consistent with earlier studies showing that post-injury immune challenge can worsen the functional post-TBI outcome. For example, LPS administration at 30 days post-TBI exacerbated cognitive impairment and induced depression-like behavior, both of which were associated with microglial reactivation and an exaggerated production of pro-inflammatory cytokines IL1-β and TNFα [45,46].

Such as in humans, brain injury caused by TBI in adult rodents presents differently between animals, even when the impact force is comparable [47]. In addition, the progression of injury varies: smaller lesions with a perilesional inflammatory rim in approximately 50% of rats and fast-progressing cortical lesions with an extensive loss of cortical tissue and large ventricle size in another 50% [47,48]. The inter-animal variability confirmed in the present study allowed us to compare the effect of a second hit induced by LPS treatment according to the lesion type. Interestingly, rats with smaller focal lesions and a perilesional inflammatory rim developed a greater response to PTZ test than animals with large cortical lesions. This correlates with our previous functional MRI study, indicating perilesional focal seizure onset following PTZ-administration in rats with a TBI_FI_ endophenotype on the basis of the blood-oxygen-level-dependent (BOLD) response [27]. Overall, these data suggest a greater presence, and consequently, a more extensive focal reactivation of the immune cells by a “second hit” in rats with the TBI_FI_ endophenotype.

### 4.3. TBI-Induced Perilesional Cortical Neuronal c-Fos Expression Was Augmented by LPS Treatment

Expression of c-Fos immunoreactivity is widely used as a biologic marker of neuronal activation following various stimuli. Both seizure and injury effects are reported. After PTZ administration, the increase in the brain c-Fos expression peaks at 2 h [49]. Here, we analyzed c-Fos expression to map the PTZ-induced spatial distribution and density of neuronal activation in the perilesional cortex and hippocampus in injured and sham-operated rat brain with or without exposure to LPS treatment at 2 h after convulsant exposure. As expected, we found a clear injury effect on excitability as the neuronal c-Fos activation to the convulsant challenge was substantially greater in rats with TBI as compared to sham-operated experimental controls. In addition, we found an infection effect on c-Fos levels: in non-infected animals, the augmented c-Fos expression was ipsilateral, whereas in LPS-treated animals, not only was the ipsilateral activation greater, but the contralateral cortex was also activated. Laminar analysis of c-Fos activation revealed greater activation in layers II-IV than in layers V-VI in both the TBI-Veh and TBI-LPS groups, indicating the contribution of the supragranular layers to the ictogenic network.

Our observations of the injury effect on c-Fos expression are consistent with previous studies in various TBI models. Following penetrating brain injury, induction of *c-fos* mRNA and protein is focal and restricted to the ipsilateral hemisphere [50]. In controlled cortical impact injury and lateral FPI models, *c-fos* mRNA expression increases in the ipsilateral cortex [51,52]. In addition to injury type, impact severity affects the distribution of c-Fos activation. [52]. Raghupathi et al. reported that mild TBI triggered by lateral FPI induced *c-fos* mRNA in the injured hemisphere, while moderate injury-induced *c-fos* mRNA bilaterally [53].

The functional significance of *c-fos* induction after TBI remains to be investigated. However, the protein product of *c-fos* forms a heterodimer with c-JUN, which binds to the AP-1 DNA site and regulates the function of multiple targets, encoding enzymes, receptors, growth factors or structural proteins, and can contribute to the remodeling of neuronal circuits within the lesioned area, eventually leading to PTE [54].

### 4.4. TBI-Induced Neuronal c-Fos Expression in the Dentate Gyrus Was Augmented by LPS Treatment

Such as in the cerebral cortex, an injury effect on c-Fos expression in the dentate gyrus has been described both in lateral FPI and cortical contusion injury models [51,52]. Some studies also demonstrated an injury-induced increase in c-Fos expression in the dentate gyrus only when stage 5 generalized tonic-clonic seizures occurred in the PTZ-kindling model [37].

Our data reproduced both the injury and seizure effects on dentate gyrus c-Fos expression in the lateral FPI model. These data add to previous findings by showing that post-injury peripheral infection augmented the dentate gyrus neuronal c-Fos activation. In 2 of 11 rats in the TBI-Veh group and in 7 of 10 rats in the TBI-LPS group with stage 4–5 seizures, increased c-Fos expression was observed in the dentate gyrus. Interestingly, 1 animal in TBI-Veh group also showed enhanced dentate c-Fos expression even though no seizure developed after PTZ injection. This particular rat, however, had experienced a stage 5 spontaneous seizure at 23 h before PTZ injection. Elevated c-Fos expression was also observed in 1 rat in the TBI_CF_-LPS group that developed a stage 3 seizure in the PTZ test. Thus, 80% of rats in the TBI-LPS group and 27% in the TBI-Veh group exhibited neuronal activation in the dentate gyrus, showing augmentation of the dentate gyrus response to convulsant challenge in rats with post-TBI infection. In particular, rats with the TBI_FI_ cortical lesion endophenotype showed robust bilateral dentate gyrus activation to PTZ challenge.

## 5. Conclusions

In conclusion, our data provide the first evidence that peripheral infection at a chronic post-TBI time-point enhances neuronal excitability in the perilesional cortex and bilaterally in the dentate gyrus, particularly in animals with prolonged focal cortical T2 enhancement around the lesion core. Our results emphasize the need for careful diagnosis and treatment of peripheral infection after TBI as a component of antiepileptogenesis treatment strategies.

## Figures and Tables

**Table 1:** Electroencephalograhic (EEG) events in the pentylenetetrazol (PTZ) seizure susceptibility test at 23 weeks after traumatic brain injury (TBI) and 15 weeks after lipopolysaccharide (LPS) administration in the whole animal group.

Parameter	Sham (1/5)	TBI + Veh (5/11)	TBI + LPS (8/10)
latency to the first spike (s)	287 ± 230	730 ± 765	374 ± 401
latency to the first ED (s)	288 ± 229	775 ± 802	400 ± 399
occurrence of PTZ-induced seizures	20%	46%	80%
latency to the first electrographic seizure (s)	1 628	604 ± 345	331 ± 258
mean seizure duration per rat (s)	24	35 ± 19	114 ± 53 *
mean cumulative seizure duration per rat (s)	24	35 ± 19	163 ± 90 **
mean behavioral seizure score per rat	3	3.2 ± 2.0	4.0 ± 1.0

Abbreviations: ED, epileptiform discharge;TBI, traumatic brain injury; Veh, vehicle. Data are shown as mean ± standard deviation of the mean. Statistical significances: * *p* < 0.05; ** *p* < 0.01 (Mann–Whitney U test compared to the TBI-Veh group).

## Supplementary Materials

The following are available online https://www.mdpi.com/article/10.3390/biomedicines9121946/s1. Figure S1: c-Fos expression in the rostromedial and rostrolateral perilesional cortex and corresponding contralateral cortex. Figure S2: Density of c-Fos expression in layers II-IV (supragranular layers) and layers V-VI (infragranular layers) in the rostral perilesional cortex. Figure S3: Representative higher magnification photomicrographs showing c-Fos immunochemistry and Nissl staining in the rostromedial contralateral cortex. Figure S4: Representative high magnification photomicrographs showing c-Fos immunochemistry and Nissl staining in the caudomedial contralateral cortex.
