# Supplementary figures and images for: Peripheral Infection after Traumatic Brain Injury Augments Excitability in the Perilesional Cortex and Dentate Gyrus
Source: Biomedicines. 2021 Dec 19;9(12):1946. doi: 10.3390/biomedicines9121946 (PMC8698476; doi:10.3390/biomedicines9121946)

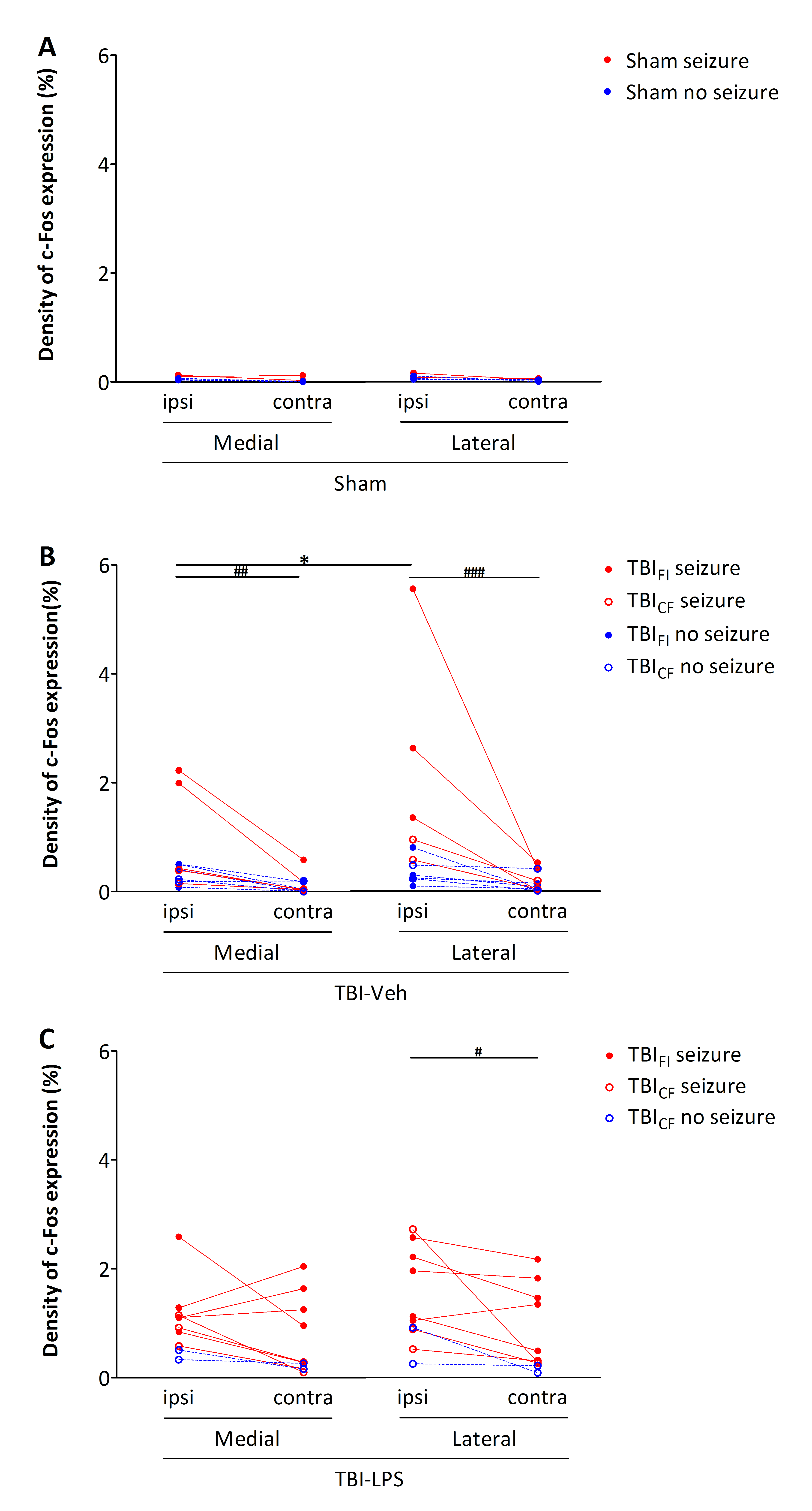

Supplement: Supplementary file 1 [file biomedicines-09-01946-s001.zip › supplementary files/Supplementary Figure S1.tif]

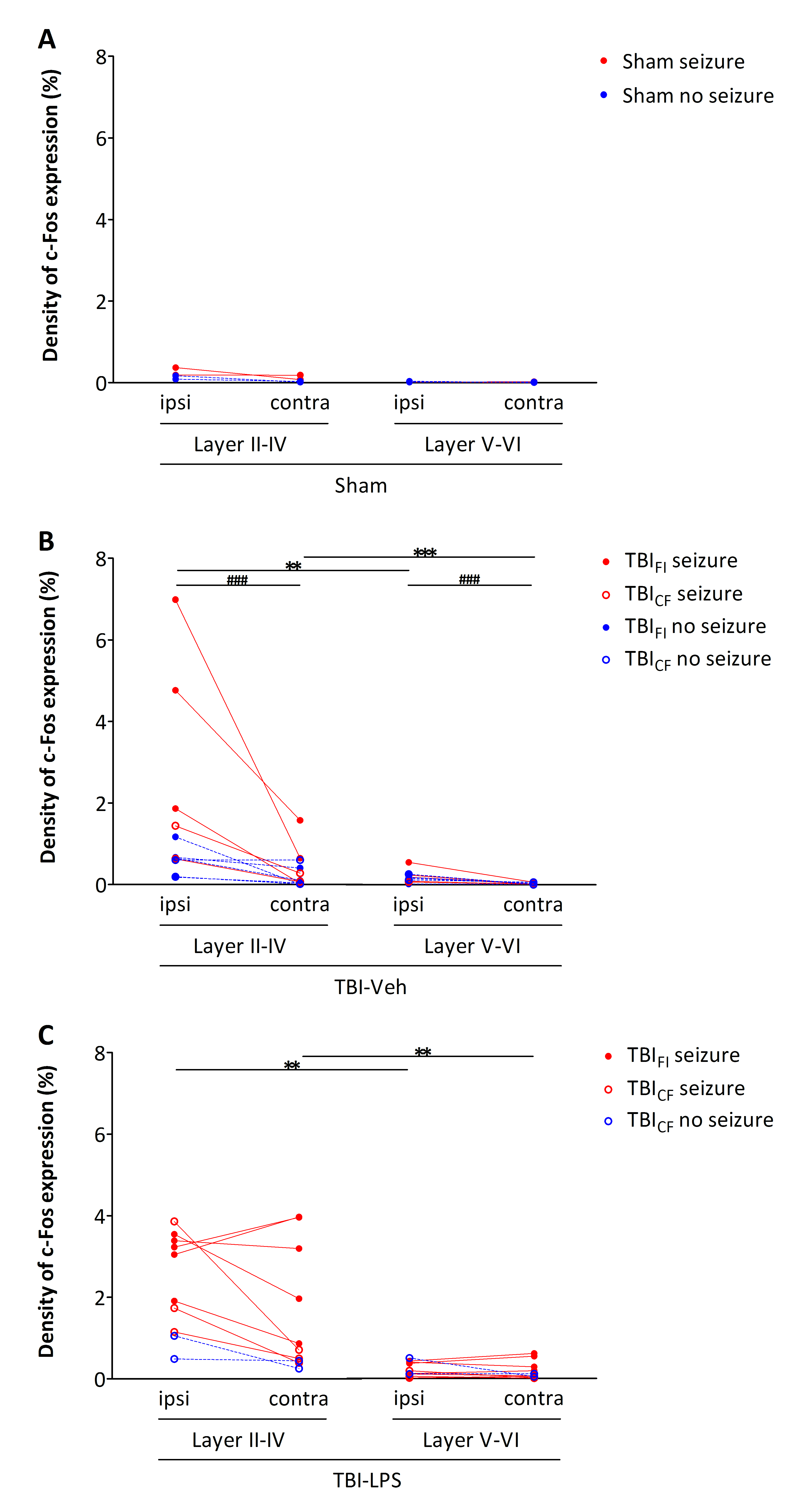

Supplement: Supplementary file 1 [file biomedicines-09-01946-s001.zip › supplementary files/Supplementary Figure S2.tif]

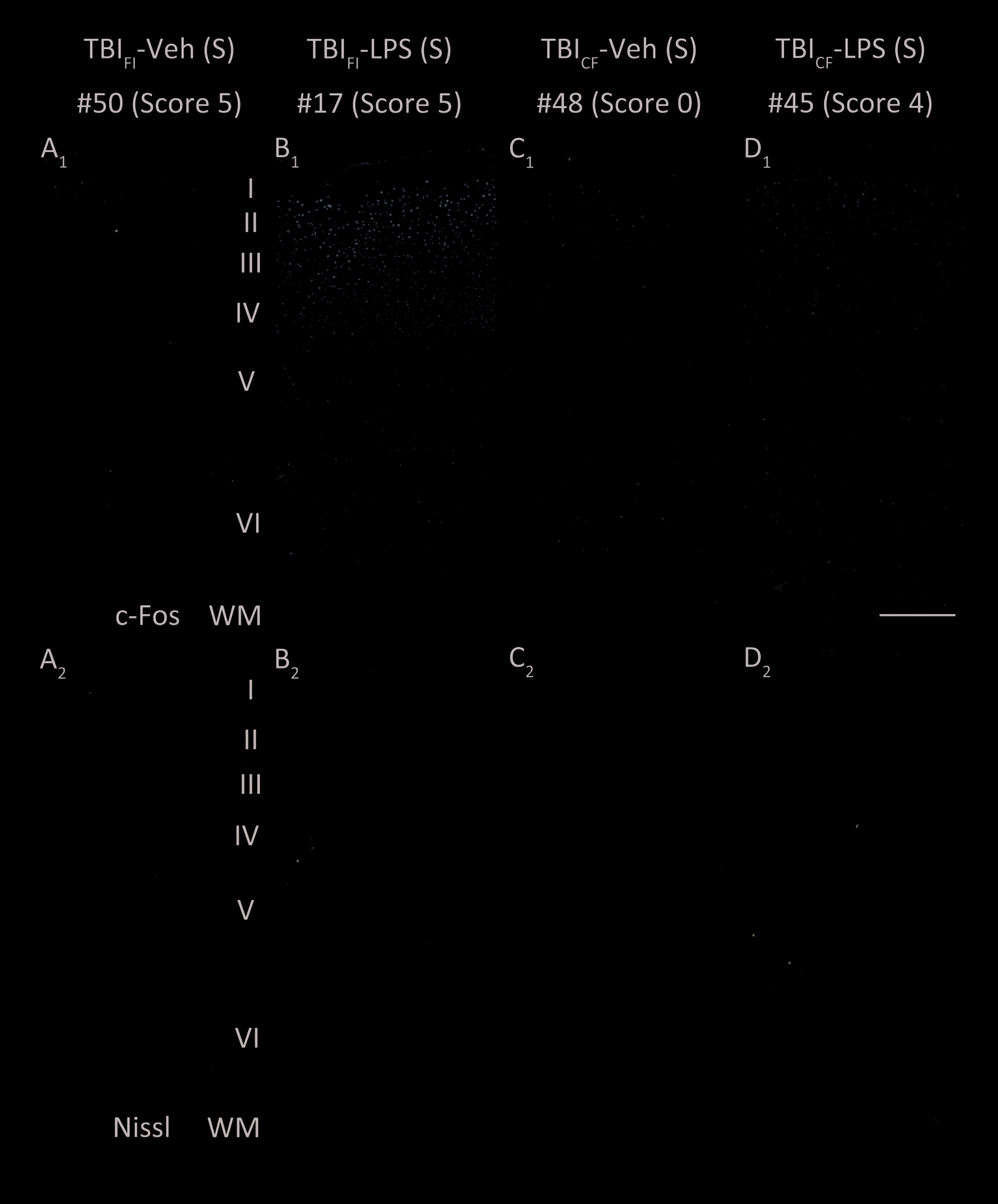

Supplement: Supplementary file 1 [file biomedicines-09-01946-s001.zip › supplementary files/Supplementary Figure S3.tif]

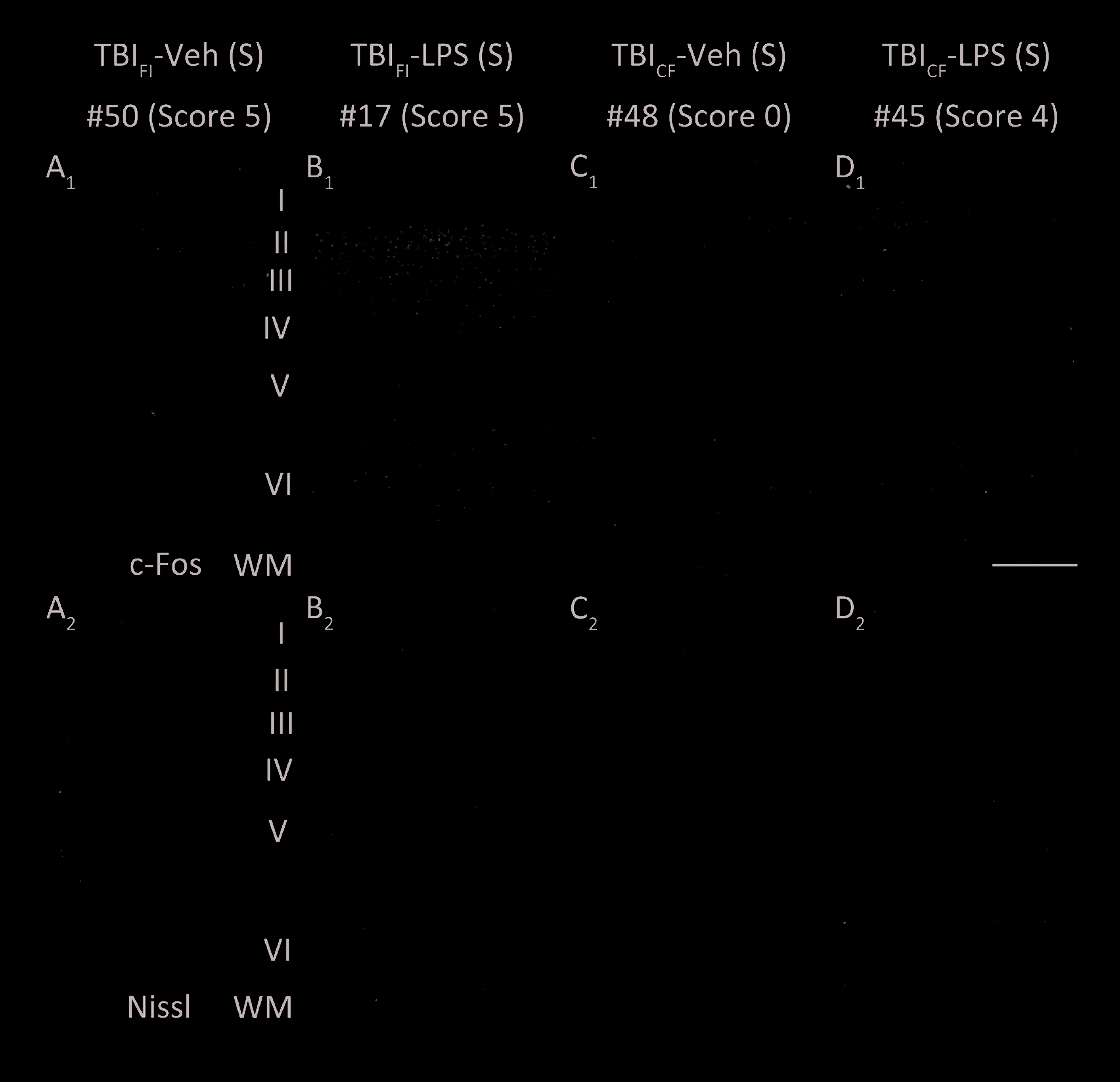

Supplement: Supplementary file 1 [file biomedicines-09-01946-s001.zip › supplementary files/Supplementary Figure S4.tif]
